# MDM4 enables efficient human iPS cell generation from PBMCs using synthetic RNAs

**DOI:** 10.1038/s41598-025-16446-y

**Published:** 2025-09-08

**Authors:** Masato Nakagawa, Mizuho Nogi, Hatsuki Doi, Ryuhei Hayashi, Tomohiko Katayama, Hirohisa Ohno, Megumi Mochizuki, Karin Hayashi, Hirohide Saito

**Affiliations:** 1 Center for iPS Cell Research and Application (CiRA), Kyoto University, Kyoto, 606-8507 Japan; 2 Premium Research Institute for Human Metaverse Medicine (WPI-PRIMe), The University of Osaka, Osaka, 565-0871 Japan; 3 Department of Ophthalmology, Graduate School of Medicine, The University of Osaka, Suita, Osaka 565-0871 Japan; 4 Department of Stem Cells and Applied Medicine, Graduate School of Medicine, The University of Osaka, Suita, Osaka 565-0871 Japan; 5 Institute for Quantitative Biosciences, The University of Tokyo, Tokyo, 113-0032 Japan

**Keywords:** Reprogramming, Induced pluripotent stem cells

## Abstract

If iPS cells can be established easily and efficiently using freshly collected blood cells, it will enhance regenerative and personalized medicine. While reports of iPS derivation from blood-derived endothelial progenitor cells using RNA have been documented, none have been reported from peripheral blood-derived mononuclear cells (PBMCs). In this study, we established a method to generate iPS cells from PBMCs using synthetic RNAs and found that MDM4, which suppresses p53, improved reprogramming efficiency.

## Introduction

Recently, the development of regenerative medicine and cell therapy using induced pluripotent stem (iPS) cells has gained significant momentum. Additionally, valiant efforts to elucidate disease states and discover new therapeutic drugs with patient-derived iPS cells are evident^1–5^. More recently, attempts have been made to create individual iPS cells tailored for personalized medicine. Consequently, there is an increasing demand for more efficient production of high-quality iPS cells.

Human iPS cells were first established from skin-derived fibroblasts (human dermal fibroblasts, HDFs) on feeder cells using retroviruses^6^. The method was stable enough to produce iPS cells, making it suitable for research. However, many challenges had to be addressed when considering future applications for iPS cell-based cell therapy and regenerative medicine. The world’s first clinical-grade iPS cells were produced from human peripheral blood-derived mononuclear cells (PBMCs) using plasmid vectors under feeder-free culture conditions^7^. Since there is a risk that plasmid vectors may integrate into the genomic DNA of iPS cells generated via this method, an approach avoiding the insertion of exogenous genes has become necessary. Currently, the most efficient method for generating iPS cells involves using Sendai virus^8^. However, it is crucial to consider the biosafety level requirements and restrictions on vector modification as potential limitations when utilizing Sendai virus vectors.

Meanwhile, researchers have reported that synthetic RNA, including messenger RNA (mRNA) and microRNA (miRNA), can be used to create iPS cells^9,10^. The transfected RNA does not remain in the cells, and the ability to synthesize any RNA with the desired sequence in vitro represents a significant advantage. Although synthetic RNA has been utilized to generate iPS cells from human fibroblasts and blood-derived endothelial progenitor cells, their generation from PBMCs has yet to be reported^11^. While recent reports, including conference abstracts^12^, have described PBMC reprogramming using RNA methods, detailed peer-reviewed data are scarce, and the molecular mechanisms remain poorly understood. In this study, we generated iPS cells from PBMCs using synthetic RNA and found that MDM4, which suppresses p53 function, significantly increased the reprogramming efficiency.

**Fig. 1:**
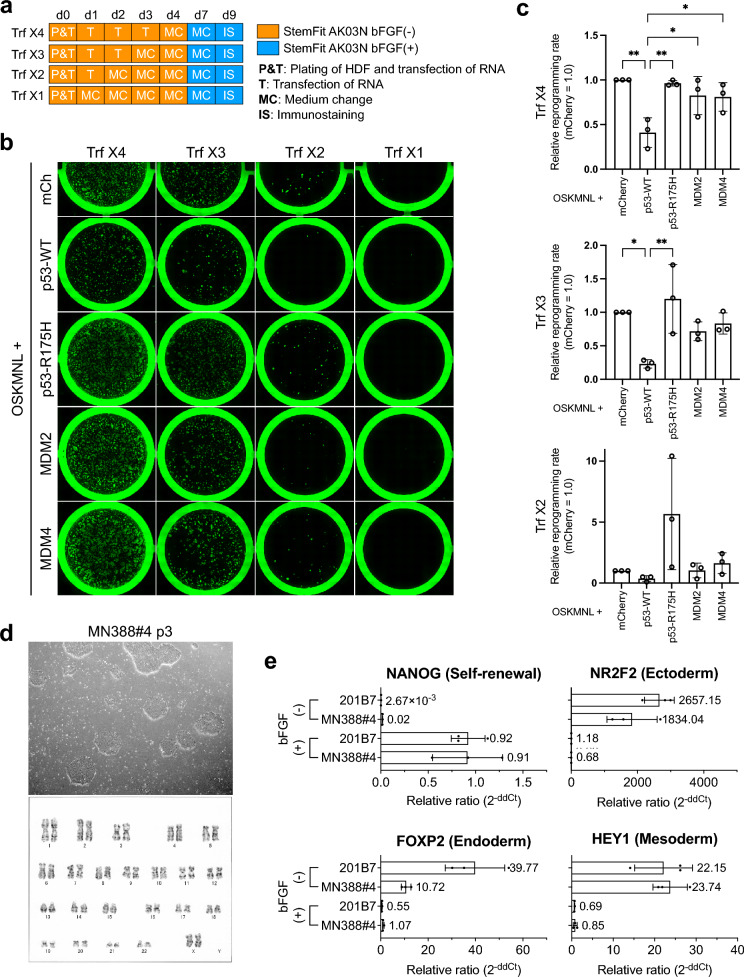
Generation of iPS cells from HDFs using synthetic RNA (**a**). Schematic diagram of HDF reprogramming experiments using synthetic RNA. On day 0 (d0), HDFs were harvested and seeded together with the required number of cells mixed with media (StemFit AK03N without bFGF), coating substrate (iMatrix-511), and synthetic RNA transfection solution. The transfection of synthetic RNA from day 1 to day 3 was performed by replacing the old medium with a fresh medium containing the newly prepared synthetic RNA transfection solution. The medium was changed to StemFit AK03N containing bFGF on day 7, with TRA-1–60 immunostaining performed on day 9. (**b**). Representative images of TRA-1–60 immunostaining. The number of transfections is shown at the top as Trf × 1–4 (n = 3). Factors introduced with reprogramming factors (i.e., OCT3/4, SOX2, KLF4, c-MYC, NANOG, and LIN28A) (OSKMNL) are shown on the left. (**c**). Impact by the number of transfections and additional factors on reprogramming efficiency. The number of TRA-1–60-positive colonies was determined by ImageJ from the immunostaining data shown in Fig. 1b, with the reprogramming efficiency calculated from the number of HDFs used for reprogramming on day 0. Mean (SD) for n = 3 independent experiments; ∗ ∗ p < 0.01, ∗ p < 0.05, analyzed by one-way ANOVA with Tukey’s test. (**d**). Establishment of HDF-derived iPS cells using synthetic RNA. Phase-contrast image of colonies of clone MN388#4 (top) and karyotyping results (bottom). (**e**). Gene expression analysis in undifferentiated and differentiated states. RNA was extracted from cells cultured in a medium with bFGF (undifferentiated state) or without bFGF (differentiated state), with gene expression confirmed by real-time quantitative PCR using TaqMan probes (n = 3).

A protocol for reprogramming human dermal fibroblasts (HDFs) using synthetic RNA was validated with a previously described method^11^. We primarily utilized the StemRNA 3rd Gen Reprogramming Kit (REPROCELL, Japan) for this experiment. The number of synthetic RNA transfections varied from four to one. The minimum number of transfections necessary to produce iPS cells was assessed by immunostaining on day 9 (Fig. 1a). During the same experiment, we also evaluated the effects of tumor suppressor gene *TP53* and its regulatory genes (e.g., *MDM2* and *MDM4*, also known as *MDMX*) on reprogramming efficiency. Human dermal fibroblasts were cultured for four days and harvested, with the required number of cells collected. The synthetic RNA for reprogramming was mixed with the transfection reagent, combined with the cell suspension, and with iMatrix-511, a substrate for iPS cell culture, before being seeded onto a culture plate. StemFit AK03N without bFGF was the reprogramming medium until colonies appeared (up to day 7). Immunostaining was conducted on day 9, and the number of TRA-1–60-positive colonies was counted using ImageJ. The reprogramming efficiency was then calculated based on the number of HDFs seeded on day 0 (Fig. 1b and c). When *mCherry* mRNA was added to the reprogramming kit, colonies appeared to fill the entire well after four transfections. Colonies were still observed even when the number of transfections was reduced to two. However, no colonies appeared after one transfection. Since previous reports indicated that suppressing p53 functions increases reprogramming efficiency^12^, we investigated whether it could enhance RNA-mediated reprogramming. Human dermal fibroblasts were transfected with the reprogramming kit alongside p53 wild-type (WT), dominant-negative p53 (R175H^13^), MDM2, or MDM4, which are known to inhibit p53^14^. p53 WT decreased the reprogramming efficiency, while p53 R175H increased it. MDM2 and MDM4 did not affect the reprogramming efficiency of HDFs (Fig. 1b, c, and Supplemental Table 1). We established multiple iPS cell clones from HDFs using synthetic RNA. Morphological, karyotypic, and gene expression analyses were conducted on MN388#4. The results were consistent with those of previously reported iPS cells (Fig. 1d, e, and Supplemental Fig. S1).

**Fig. 2:**
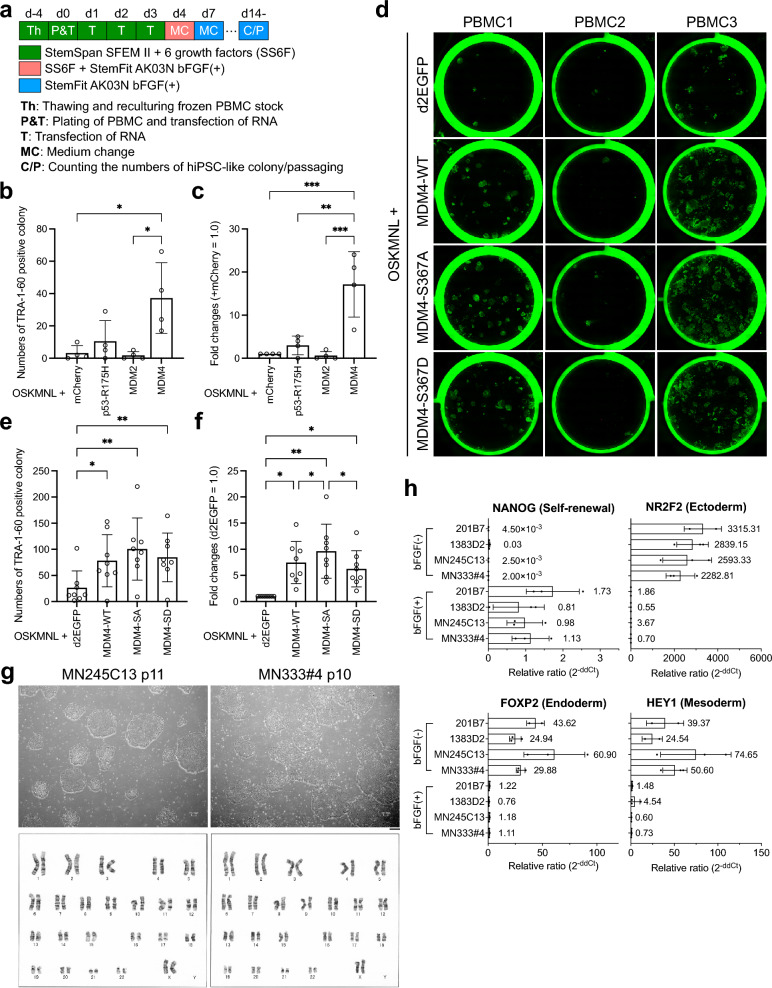
Generation of iPS cells from PBMCs using synthetic RNA (**a**). Schematic diagram of PBMC reprogramming experiments using synthetic RNA. On day −4 (d-4), frozen stocks were thawed and incubated in the PBMC culture medium (SS6F). On day 0 (d0), PBMCs were harvested and seeded together with the required number of cells mixed with media (SS6F), coating substrate (iMatrix-511), and synthetic RNA transfection solution. The transfection of synthetic RNA from day 1 to day 3 was performed by replacing the old medium with a fresh medium containing the newly prepared synthetic RNA transfection solution. The medium was changed to a mix of SS6F and StemFit AK03N containing bFGF on day 4 and StemFit AK03N containing bFGF on day 7, with TRA-1–60 immunostaining performed on day 14. (**b**) and (**c**). Effect of dominant-negative p53 and MDMs on PBMC reprogramming. The number of TRA-1–60-positive colonies was determined using ImageJ from the immunostaining data (**b**), and the reprogramming efficiency was calculated based on the number of PBMCs used for reprogramming on day 0 (n = 4). Relative reprogramming efficiencies were normalized to PBMCs transfected with *mCherry* mRNA as the additional factor (**c**). Mean (SD) for n = 4 independent experiments; ∗ ∗ ∗ p < 0.001, ∗ ∗ p < 0.01, ∗ p < 0.05, analyzed by one-way ANOVA with Tukey’s test. (**d**). Representative images of TRA-1–60 immunostaining. Eight lots of PBMCs were used in this experiment (n = 8; PBMC lot information is shown in Supplemental Table 2). This figure shows immunostaining results from three lots of PBMCs (PBMC1-3, lot numbers are listed in Supplemental Table 2). Factors introduced together with OSKMNL reprogramming factors are shown on the left. (**e**) and (**f**). Effect of MDM4s on reprogramming efficiency. The number of TRA-1–60-positive colonies was determined by ImageJ from the immunostaining data shown in Fig. 2 d (**e**), with the reprogramming efficiency calculated from the number of PBMCs used for reprogramming on day 0 (**f**). Mean (SD) for n = 8 independent experiments; ∗ ∗ p < 0.01, ∗ p < 0.05, analyzed by one-way ANOVA with Tukey’s test.

Next, we examined whether we could produce iPS cells from PBMCs using synthetic RNA. While the method and frequency of RNA transfection were identical to those used for reprogramming HDF, the culture medium used was optimized for PBMCs (Fig. 2a). iPS cell-like colonies of sufficient size emerged approximately 14 days after the initial RNA transfection, at which point immunostaining was conducted. When reprogramming PBMCs, only a limited number of colonies appeared after adding *mCherry* mRNA to the reprogramming kit, but the quantity increased slightly with the introduction of p53 R175H. By contrast to HDF, *MDM4* mRNA significantly enhanced the efficiency of PBMC reprogramming (Fig. 2b and c). MDM4 serine 367 phosphorylation is known to lead to degradation via the ubiquitin pathway. The mutant S367A (MDM4-SA), with serine replaced by alanine, has been reported to be resistant to degradation^15^. We thus investigated the impact of this mutant on PBMC reprogramming (Fig. 2d, e, and f). Among the three samples of PBMCs presented in Fig. 2d, MDM4-S367A yielded the highest number of TRA-1–60-positive colonies. Notably, in the case of PBMC lot 2, which exhibited poor reprogramming efficiency, almost no colonies were observed except when MDM4-S367A was involved (Fig. 2e, f, and d, along with Supplemental Table 2). We also assessed the effect of the MDM4-S367D (MDM4-SD) mutant, which mimics the phosphorylated state, but its reprogramming ability was comparable to that of MDM4-WT. Although we observed that the S367A mutant of MDM4 appeared to exhibit the highest enhancement of reprogramming efficiency, this conclusion is based on experiments using PBMCs derived from eight independent donors. While statistical analysis indicated a significant difference compared to other MDM4 variants, the absolute magnitude of this difference was modest, as shown in the graph. Therefore, we acknowledge that the current data do not provide definitive proof of the superior efficacy of the S367A mutant and that further investigation will be necessary to confirm its mechanistic advantage. iPS cell clones established using synthetic RNA from PBMCs exhibited normal colony morphology and karyotype (Fig. 2g), with gene expression similar to that of a well-established iPS cell line (Fig. 2h and Supplemental Fig. S2). To evaluate the differentiation potential of PBMC-derived iPSCs generated via synthetic RNA, we subjected the MN328 clone to corneal epithelial differentiation using the SEAM protocol^13–16^. After sequential culture in differentiation, corneal specification, and maturation media over a 10–11-week period, we observed morphological changes indicative of epithelial commitment under phase-contrast microscopy (Supplemental Fig. S3a). Immunostaining at 4 weeks of differentiation culture showed co-expression of PAX6 and p63, consistent with corneal epithelial progenitor identity (Supplemental Fig. S3b). We further isolated CD200⁻ITGB4⁺SSEA4⁺ cells by FACS, which were then expanded on iMatrix-511-coated inserts. The resulting epithelial sheets exhibited uniform expression of PAX6, p63, and KRT12, demonstrating successful differentiation into corneal epithelial-like cells (Supplemental Figs. S3c and S3d). These findings highlight the capacity of PBMC-iPSCs to generate clinically relevant somatic cell types.

In this study, we successfully used synthetic RNA to generate iPS cells from HDFs and PBMCs isolated from human blood. Notably, our work revealed the critical role of MDM4, which inhibits p53, in conjunction with other reprogramming factors when utilizing blood cells. The initial step in establishing a synthetic RNA-based reprogramming protocol involved mixing all cells, culture medium, synthetic RNA, and coating substrate, then seeding them together during the initial transfection. This approach is believed to enhance efficiency compared to introducing RNA into adherent cells, as RNA can be delivered from the entire cell surface.

Suppressing p53 functions is known to increase reprogramming efficiency^12,16–18^. As such, dominant-negative mutants of p53 and siRNA targeting p53 are routinely employed. Once again, the dominant-negative mutant of p53 had the most significant effect when human dermal fibroblasts (HDFs) were reprogrammed using synthetic RNA. MDM4, as well as MDM2, is well known to suppress the function of p53. However, both MDM2 and MDM4 had little effect on HDF reprogramming using synthetic RNA. By contrast, MDM4 exhibited the most substantial effect when reprogramming PBMCs, indicating that the reprogramming mechanism with synthetic RNA varies slightly between HDFs and PBMCs. MDM4 may serve additional roles in PBMC reprogramming beyond suppressing p53 function. MDM4 is traditionally recognized as a negative regulator of p53 transcriptional activity; however, its precise role in promoting reprogramming, particularly in stress-sensitive PBMCs, remains incompletely understood. Our findings suggest that MDM4 preferentially enhances reprogramming efficiency in PBMCs but not in HDFs, possibly due to intrinsic differences in p53 pathway activation. PBMCs, which are largely quiescent in vivo, exhibit heightened sensitivity to stress-induced p53 activation—such as that triggered by synthetic mRNA transfection—whereas HDFs display a more muted response. We propose that MDM4 suppresses this transient p53 surge in PBMCs, thereby preventing early apoptosis and supporting reprogramming progression. Moreover, the S367A mutant of MDM4, which is resistant to ubiquitin-mediated degradation^17^, may prolong p53 inhibition during critical early stages. Beyond its canonical function, MDM4 has also been reported to interact with components of the cell cycle and DNA repair machinery, including the ATR-Chk1 axis^18^, suggesting that it may help establish a reprogramming-permissive state by modulating broader stress responses and cell cycle dynamics. We aim to clarify this in future research and develop more efficient reprogramming methods.

Although Tang et al. (2025) reported the use of mRNA for PBMC reprogramming, their data were presented only in a conference abstract without experimental details^12^. Likewise, no group at the ISSCR2025 meeting demonstrated reproducible and quantitative RNA-based reprogramming from PBMCs with clonal validation and mechanistic insight, as we report here. This study presents the first comprehensive report on the synthetic RNA-based reprogramming of PBMCs, supported by systematic functional validation and mechanistic analysis.

The results of this study enabled the production of iPS cells from PBMCs isolated from blood. This development will significantly contribute to cell therapy and regenerative medicine using iPS cells. Research and development of personalized medicine utilizing individual iPS cells has gained momentum recently. Because blood samples can be acquired with relative ease and minimal invasiveness, our findings will prove invaluable in further advancing iPS cell-based personalized medicine. Separating PBMCs from a blood sample takes approximately an hour, and mRNA transfection is safe due to its short half-life and lack of genomic integration^19^. Thus, PBMCs represent a promising cell resource for generating iPS cells with mRNA for clinical applications. The immediate availability of blood samples for iPS cell establishment after collection offers a notable advantage.

MDM4-WT: MDM4 wild-type, MDM4-SA: MDM4 S367A, MDM4-SD: MDM4 S367D. (**g**). Establishment of PBMC-derived iPS cells using synthetic RNA. Phase-contrast image of colonies of clones MN245C13 and MN333#4 (top) and karyotyping results (bottom). (**h**). Gene expression analysis in undifferentiated and differentiated states. RNA was extracted from cells cultured in a medium with bFGF (undifferentiated state) or without bFGF (differentiated state), with gene expression confirmed by real-time quantitative PCR using TaqMan probes (n = 3).

## Methods

### *Synthesis of mRNA *in vitro

mCherry, d2EGFP, p53 WT, p53 R175H, MDM2 WT, MDM4 WT, MDM4 S367A, and MDM4 S367D cDNAs were amplified by PCR and subcloned into plasmid vectors (T7 promoter) for in vitro RNA synthesis. We used a kit (NIPPON GENE, CUGA 7 in vitro Transcription kit, 307–13531) to synthesize mRNA and followed the attached protocol. CleanCap Reagent AG (3’OMe) (TriLink, N-7413–1) was used as a capping reagent. The Monarch RNA Cleanup Kit (NEW ENGLAND Biolabs, T2040S) was used to purify the synthesized RNA, and a Qubit 4 Fluorometer (Invitrogen, Q33238) was used to measure its concentration and purity.

### HDF reprogramming

Frozen HDF stocks (Cell Applications, INC., CA10605f) were thawed and cultured in DMEM (Nacalai Tesque, 08459–64) containing 10% FBS (SIGMA, F7524) for 4 days. Cells were harvested with trypsin (Gibco, 25200056), and the required number of cells were prepared in StemFit AK03N medium (AJINOMOTO, SF010-002) without bFGF (StemFit AK03N (-)) at a concentration of 5.0 × 10^5^ cells/mL (day 0). The StemRNA 3rd Gen Reprogramming Kit (REPROCELL, 00–0076) was used for reprogramming. The transduction method followed the protocol attached to the kit. For reprogramming, 5.0 × 10^4^ cells were seeded in one well of a 24-well plate. A total of 200 ng of mRNA encoding OSKMNL (0.76 μL), EKB (0.67 μL), and the additional factors synthesized in our lab (0.13 μL, 100 ng/mL), and microRNAs (0.16 μL) was mixed with 0.72 μL of Lipofectamine MessengerMAX Transfection Reagent (Invitrogen, LMRNA001) as the RNA transfection solution (RNAs and MessengerMAX were diluted with Opti-MEM medium (Invitrogen, 31985062)). A cell suspension (100 μL), RNA transfection solution, iMatrix-511 (1.3 μL) (MATRIXOME, 892–011), and StemFit AK03N (-) medium were mixed (total volume, 800 μL) and seeded into a well immediately. From day 1 to day 3, synthetic RNA was introduced as a medium and transfection solution mixture. The medium was changed to StemFit AK03N (-) on day 4 and StemFit AK03N containing bFGF (StemFit AK03N (+)) on day 7. Cells were immunostained with an anti-TRA-1–60 antibody or passaged on day 9.

### PBMC reprogramming

Frozen PBMC stocks (Cellular Technology Ltd, CTL-UP1) were thawed and cultured in StemSpan SFEM II (STEMCELL Technologies, ST-09605) supplemented with six cytokines (20 ng/mL IL-3 (090–05761), 50 ng/mL IL-6 (098–06041), 10 ng/mL TPO (207–17581), 20 ng/mL Flt3L (061–05391), 50 ng/mL SCF (197–15511), and 10 ng/mL G-CSF (072–06101) (Fujifilm Wako Chemicals)) (SS6F medium) for 4 days. Cells were harvested after trypsinization, and the required number of cells was prepared in SS6F medium at a concentration of 1.0 × 10^6^ cells/mL (day 0). The StemRNA 3rd Gen Reprogramming Kit was used for reprogramming. For reprogramming, 1.0 × 10^5^ cells were seeded in one well of a 24-well plate. A total of 400 ng of mRNA encoding OSKMNL (1.48 μL), EKB (1.33 μL), and the additional factors synthesized in our lab (0.3 μL, 100 ng/mL), and microRNAs (0.31 μL) was mixed with 1.44 μL of Lipofectamine MessengerMAX Transfection Reagent as the RNA transfection solution (RNAs and MessengerMAX were diluted with Opti-MEM medium). A cell suspension (100 μL), RNA transfection solution, iMatrix-511 (2.5 μL), and SS6F medium were mixed (total volume, 800 μL) and seeded into a well immediately. From day 1 to 3, synthetic RNA was introduced as an SS6F medium and transfection solution mixture. The medium was changed to a mixture of SS6F medium and StemFit AK03N (+) (mix ratio 3:2) on day 4 and StemFit AK03N (+) on day 7. Cells were immunostained with an anti-TRA-1–60 antibody or passaged on day 14. PBMCs used in this study were obtained from eight independent healthy donors with diverse demographic backgrounds, including ethnicity, age, gender, and blood type (Supplemental Table 2). Each donor-derived PBMC sample was reprogrammed independently, and the enhancement of reprogramming efficiency by MDM4 was consistently observed across all donors, demonstrating the robustness of the approach.

### Immunostaining and colony counting

Reprogrammed cells were fixed with a 4% paraformaldehyde phosphate buffer solution (Fujifilm Wako Chemicals, 163–20145) for 10 min at RT. Fixed cells were stained with an anti-TRA-1–60 antibody (BD, 560071) followed by an anti-mouse Alexa488 antibody (Invitrogen, A21042). Fluorescent images of the entire wells were acquired using a microscope system (Keyence, BZ-710), and colony numbers were counted using ImageJ software.

### iPS cell culture

Cultures for maintaining pluripotency or differentiating iPS cells were performed according to previous methods^7,19^. Human iPS cell lines were cultured on 0.5 μg/cm^2^ iMatrix-511 in StemFit AK03N containing bFGF for 7 days. TrypLE Select Enzyme (12563011, Gibco) was used to detach and dissociate cells. Cell numbers were counted using Countess 3 (Invitrogen, AMQAX200). After mixing cells, culture medium, iMatrix-511, and Y-27632 (Rock inhibitor, 10 μM, 18188–04, Nacalai) in a single tube, the mixture was seeded onto a plate. Human iPS cells were cultured as low-density single cells by plating 2.08 × 10^3^ live cells/cm^2^. When culturing and differentiating hiPS cells without bFGF, StemFit AK03N without bFGF (solution C not included) was used when cells were collected as single cells.

### Quantification of gene expression

Gene expression was evaluated by RT-qPCR under two conditions: bFGF(+) (undifferentiated) and bFGF(-) (spontaneous differentiation). The bFGF(-) culture condition promotes spontaneous differentiation into ectoderm, mesoderm, and endoderm lineages, as demonstrated in previous work^7^. Total RNA was purified, and RT-qPCR was performed as described previously^7^. Quantitative real-time PCR was performed using TaqMan Gene Expression Assays (Applied Biosystems) according to the manufacturer’s protocol. The probes used included NANOG (Hs02387400_g1) as a pluripotency marker; NR2F2 (Hs00819630_m1), COL2A1 (Hs00264051_m1), and ZBTB16 (Hs00232313_m1) as ectoderm markers; FOXP2 (Hs00362817_m1), CDH20 (Hs00230412_m1), and CLDN1 (Hs00221623_m1) as endoderm markers; and HEY1 (Hs01114113_m1), PDGFRA (Hs00998018_m1), and ABCA4 (Hs00979594_m1) as mesoderm markers. Relative gene expression levels were calculated using the ΔΔCt method, with normalization to the GAPDH gene.

### Karyotyping

Karyotype analysis using the G-banding method was outsourced (Special Reference Laboratories (SRL), Japan).

### Statistics

GraphPad Prism (GraphPad Software, Inc.) was used for statistical analysis. Data are presented as means ± standard deviation (SD). Unless specified otherwise, no statistically significant differences were detected.

### Corneal differentiation culture

Corneal differentiation culture (i.e., SEAM culture) was performed as described previously^14,15^. Briefly, human iPSCs (MN328) were seeded at 800 to 5,000 cells per well in 6-well plates (353046, Corning, NY, USA) coated with 0.5 μg/cm^2^ iMatrix-511 and then cultured for an additional 10 days in StemFit medium. Differentiation was initiated by culturing cells in differentiation medium (DM; GMEM (Thermo Fisher Scientific, Waltham, MA, USA)) supplemented with 10% knockout serum replacement (KSR; Thermo Fisher Scientific), 1 mM sodium pyruvate (Thermo Fisher Scientific), 0.1 mM non-essential amino acids (Thermo Fisher Scientific), 2 mM L-glutamine (Thermo Fisher Scientific), 100 units/mL penicillin potassium, and 100 μg/mL streptomycin sulfate (Meiji Seika Pharma, Tokyo, Japan) and 55 μM monothioglycerol (Wako, Osaka, Japan). After 4 weeks, the medium was replaced with corneal differentiation medium (CDM; DM and CnT-PR w/o; EGF and FGF2, CELLnTEC Advanced Cell Systems, Bern, Switzerland) (1:1) containing 20 ng/mL KGF (Wako) and 10 μM Y-27632 (Wako), 100 unit/mL penicillin potassium and 100 μg/mL streptomycin sulfate) and cultured for an additional 4 weeks. The medium was then replaced with corneal epithelial differentiation medium (CEM; DMEM/F12 (Thermo Fisher Scientific) containing 2% B-27 supplement (Thermo Fisher Scientific), 20 ng/mL KGF, 10 μM Y-27632, 100 units/mL penicillin potassium and 100 μg/mL streptomycin sulfate), after which the plates were incubated for an additional 2 to 3 weeks (10 to 11 weeks in total). Phase contrast microscopy was performed using an Axio observer Z1, D1 (Zeiss, Jena, Germany).

### Cell sorting and fabrication of corneal epithelial cell sheets

The procedures were performed as described previously^13,16^. Briefly, following the corneal differentiation culture, cells were harvested using Accutase (Thermo Fisher Scientific) and stained with Alexa Fluor 647 (AF647)-conjugated anti-ITGB4 (450-9D, BD Biosciences, San Jose, CA, USA), PE-conjugated anti-SSEA-4 (MC813-70, BioLegend, San Diego, CA, USA) and PE-Cy7-conjugated anti-CD200 antibodies (OX-104, BD Biosciences). Corneal epithelial cell fraction, identified as CD200-, ITGB4 +, and SSEA4 + cells, were sorted with a FACSAriaII cell sorter (BD Biosciences) and seeded onto iMatrix-511-coated (0.5 μg/cm^2^) 6- or 12-well cell culture inserts. Cells were cultivated in CEM and corneal epithelium maturation medium (i.e., KCM^2^ (keratinocyte culture medium) containing 20 ng/mL KGF and 10 μM Y-27632) for approximately 2 weeks.

### Immunostaining

Cells were fixed in 4% paraformaldehyde (Wako) or cold acetone (Wako, for p63 staining), washed with Tris-buffered saline (TaKaRa Bio, Shiga, Japan) three times for 10 min, and incubated with Tris-buffered saline containing 5% normal donkey serum and 0.3% Triton X-100 for 1 h to block non-specific reactions. Afterwards, they were incubated with the primary antibodies (KRT12: rabbit monoclonal, clone EPR17882, ab185627, 1:200, Abcam, Cambridge, UK; p63: mouse monoclonal, clone 4A4, ab735, 1:200, Abcam; PAX6: rabbit polyclonal, PRB-278P, 901301, 1:1000, BioLegend, San Diego, CA, USA; MUC16: mouse monoclonal, clone OC125, ab693, 1:200, Abcam) at 4 °C overnight. The cells were again washed three times with Tris-buffered saline for 10 min and incubated with AF488-, AF568-, and AF647-conjugated secondary antibodies (1:200; Thermo Fisher Scientific) for 1 h at room temperature. Counterstaining was performed with Hoechst 33342 (Thermo Fisher Scientific) before fluorescence microscopy (Axio Observer D1, Zeiss).

### iPS cell lines used in this study

Here is the information on the iPS cell lines used in this study, including the cell of origin, donor age, blood type, ethnicity, sex, and reprogramming methods.

- 201B7: Human Dermal Fibroblasts, Adult (36-year), Caucasian, Female, reprogrammed using retroviral vectors (aHDF1388, Cell Applications, INC.)^6^
- 1383D2: Peripheral blood mononuclear cells, Adult (36-year, B/Pos), Asian, Male, reprogrammed using episomal plasmid vectors (HHU20061026, Cellular Technology Ltd)^7^
- MN388#4: Human Dermal Fibroblasts, Fetal (20-week gestation), Hispanic/Latino, Female (CA10605f, Cell Applications, INC.) (established in this study)
- MN245C13: Peripheral blood mononuclear cells, Adult (37-year, O/Pos), Hispanic, Female (HHU20171109, Cellular Technology Ltd) (established in this study)
- MN333#4: Peripheral blood mononuclear cells, Adult (33-year, O/Pos), Hispanic, Female (HHU20191001, Cellular Technology Ltd) (established in this study)
- MN328: Peripheral blood mononuclear cells, Adult (23-year, O/Pos), African/American, Female (HHU20190917, Cellular Technology Ltd) (established in this study)

All the iPS cell lines used in this study were generated from commercially purchased cells. No human participants were directly involved in this research.

## Supplementary Information

Supplementary Information 1.

Supplementary Information 2.

## Data Availability

The datasets used and/or analysed during the current study available from the corresponding author on reasonable request.
