# Supplementary material for: MDM4 enables efficient human iPS cell generation from PBMCs using synthetic RNAs
Source: Sci Rep. 2025 Sep 8;15:30620. doi: 10.1038/s41598-025-16446-y (PMC12417537; doi:10.1038/s41598-025-16446-y)
Supplement: Supplementary file 1 — Supplementary Information 1. [file 41598_2025_16446_MOESM1_ESM.pdf]

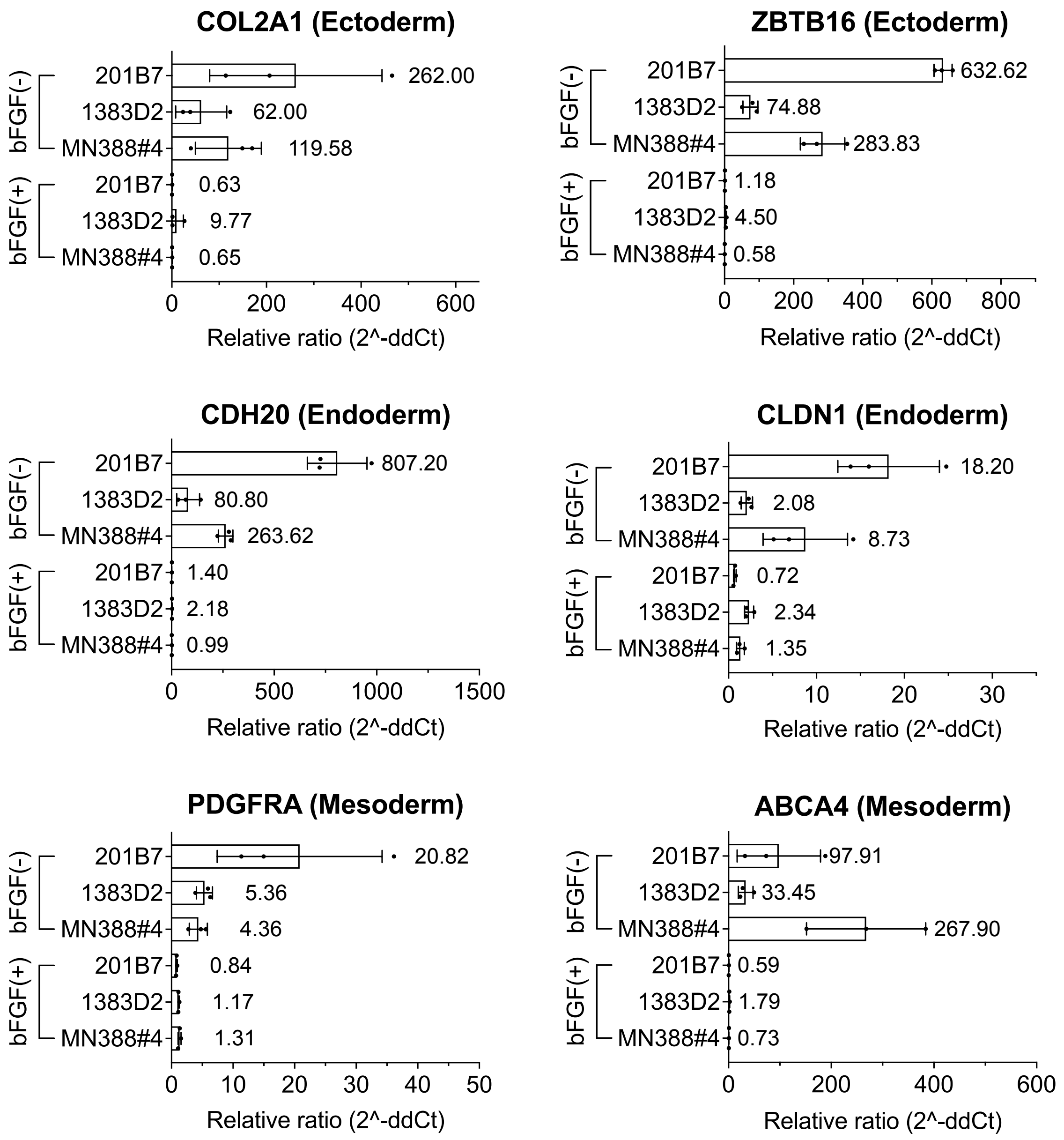
COL2A1 (Ectoderm)
bFGF(-)
bFGF(+)
201B7
1383D2
MN388#4
262.00
62.00
119.58
0.63
9.77
0.65
0 200 400 600
Relative ratio (2^-ddCt)
ZBTB16 (Ectoderm)
632.62
74.88
283.83
1.18
4.50
0.58
0 200 400 600 800
Relative ratio (2^-ddCt)
CDH20 (Endoderm)
807.20
80.80
263.62
1.40
2.18
0.99
0 500 1000 1500
Relative ratio (2^-ddCt)
CLDN1 (Endoderm)
18.20
2.08
8.73
0.72
2.34
1.35
0 10 20 30
Relative ratio (2^-ddCt)
PDGFRA (Mesoderm)
20.82
5.36
4.36
0.84
1.17
1.31
0 10 20 30 40 50
Relative ratio (2^-ddCt)
ABCA4 (Mesoderm)
97.91
33.45
267.90
0.59
1.79
0.73
0 200 400 600
Relative ratio (2^-ddCt)

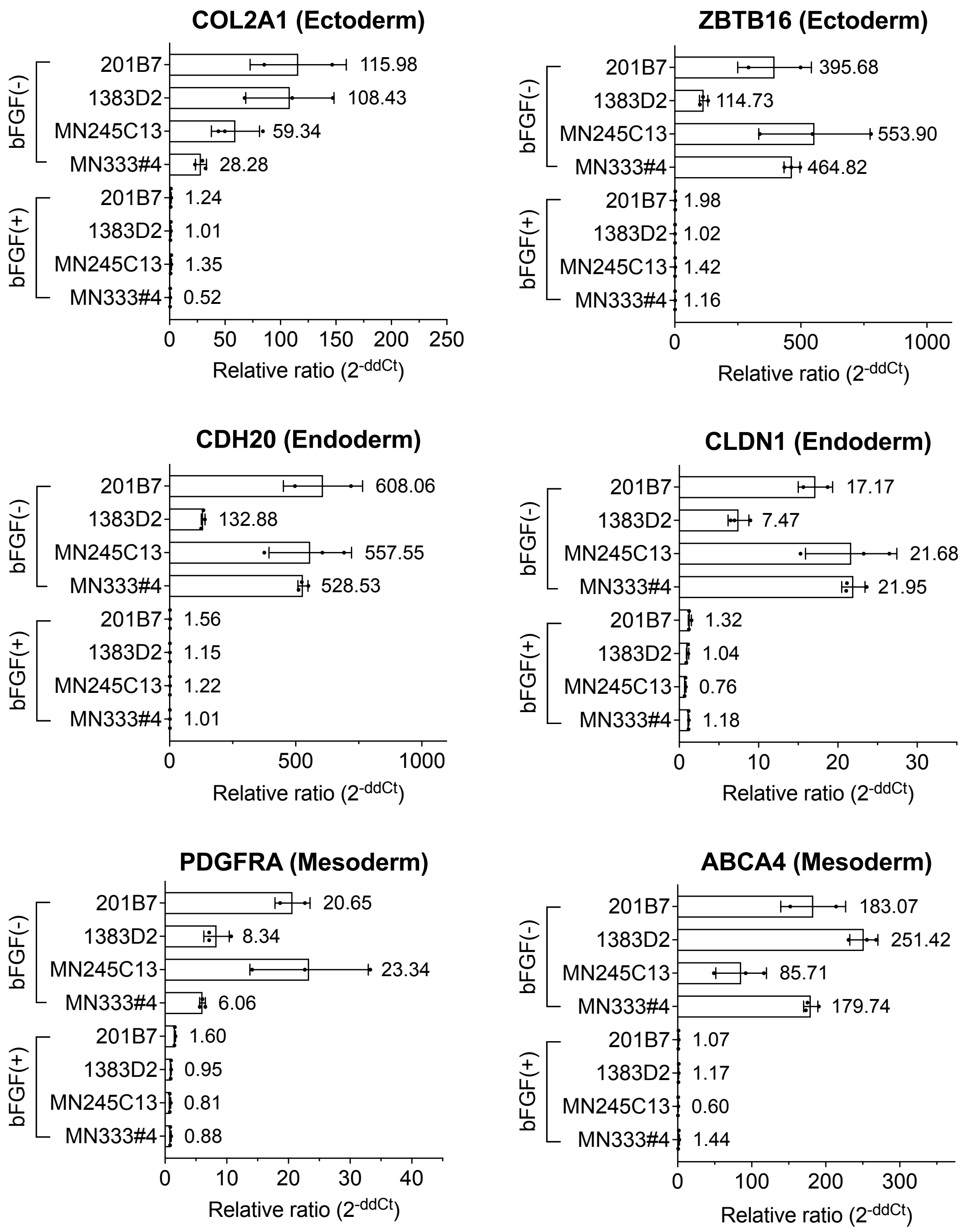
COL2A1 (Ectoderm)
bFGF(-)
bFGF(+)
201B7
1383D2
MN245C13
MN333#4
115.98
108.43
59.34
28.28
1.24
1.01
1.35
0.52
0 50 100 150 200 250
Relative ratio (2^-ddCt)
ZBTB16 (Ectoderm)
395.68
114.73
553.90
464.82
1.98
1.02
1.42
1.16
0 500 1000
CDH20 (Endoderm)
608.06
132.88
557.55
528.53
1.56
1.15
1.22
1.01
CLDN1 (Endoderm)
17.17
7.47
21.68
21.95
1.32
1.04
0.76
1.18
0 10 20 30
PDGFRA (Mesoderm)
20.65
8.34
23.34
6.06
1.60
0.95
0.81
0.88
0 10 20 30 40
ABCA4 (Mesoderm)
183.07
251.42
85.71
179.74
1.07
1.17
0.60
1.44
0 100 200 300

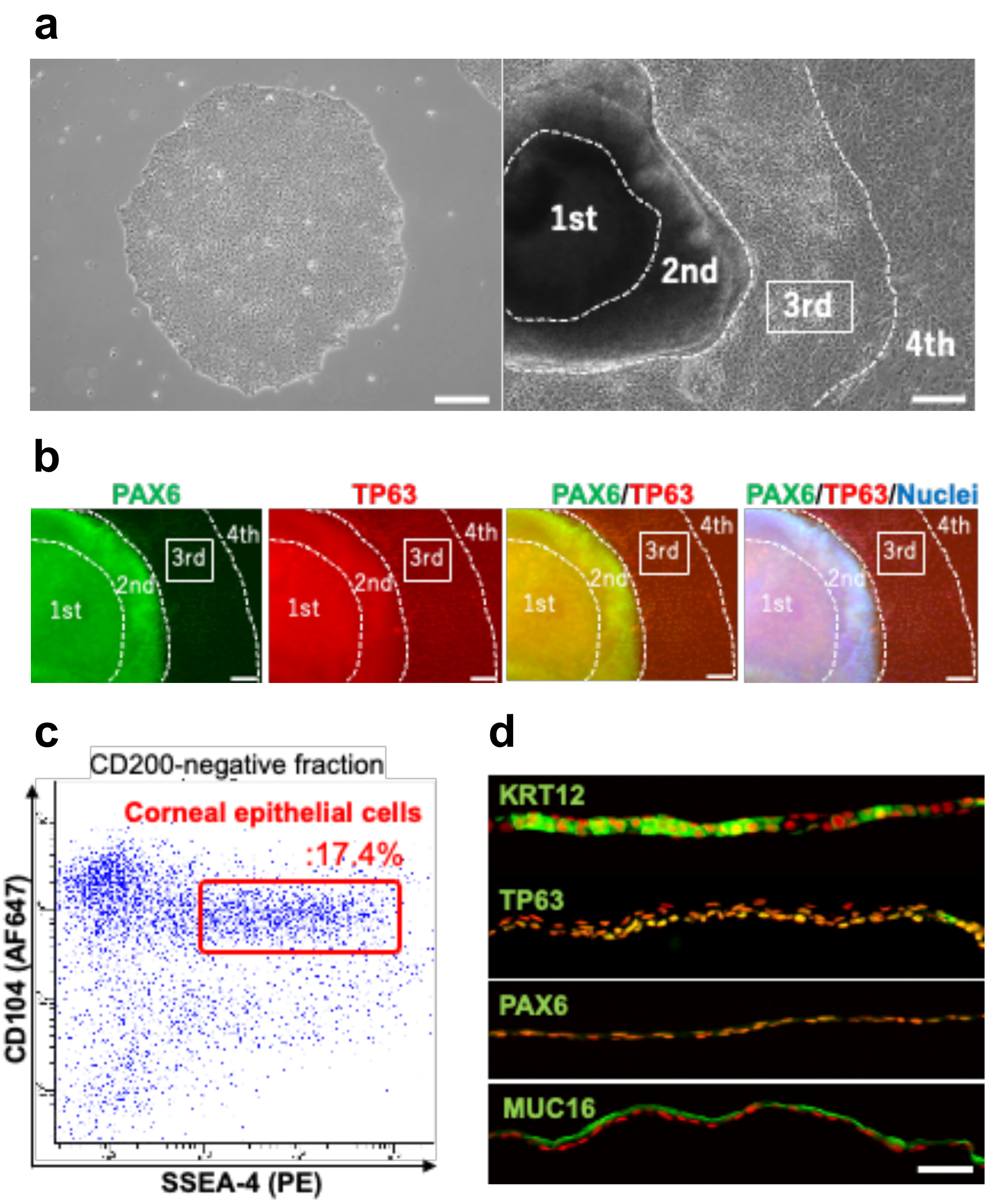

a. Phase contrast images of MN328 iPSC colony at the start (Day 0) and after 4 weeks of corneal differentiation culture. Scale bars; 200 µm
b. Immunostaining of PAX6 and p63 at 4 weeks of differentiation culture. Scale bars; 100 µm
c. Isolation of corneal epithelial cells by FACS using CD200, CD104 and SSEA-4 antibodies. A red box indicates corneal epithelial progenitors.
d. Immunostaining of corneal epithelial cell sheets prepared using corneal epithelial progenitors derived from MN328 iPSCs. Scale bar; 50 µm
