# Supplementary material for: MDM4 enables efficient human iPS cell generation from PBMCs using synthetic RNAs
Source: Sci Rep. 2025 Sep 8;15:30620. doi: 10.1038/s41598-025-16446-y (PMC12417537; doi:10.1038/s41598-025-16446-y)
Supplement: Supplementary file 2 — Supplementary Information 2. [file 41598_2025_16446_MOESM2_ESM.docx]

*Nakagawa et al.*, Supplemental Table 1

Reprogramming efficiency of HDFs using synthetic RNAs

| Trf X4 |  |  |  |  | (%) |
| --- | --- | --- | --- | --- | --- |
| Add. factor: | mCherry | p53-WT | p53-R175H | MDM2 | MDM4 |
| Exp. No.1 | 2.22 | 0.51 | 2.09 | 1.30 | 1.40 |
| Exp. No.2 | 3.12 | 1.73 | 3.11 | 2.79 | 2.66 |
| Exp. No.3 | 3.40 | 1.51 | 3.22 | 3.38 | 3.21 |

| Trf X3 |  |  |  |  | (%) |
| --- | --- | --- | --- | --- | --- |
| Add. factor: | mCherry | p53-WT | p53-R175H | MDM2 | MDM4 |
| Exp. No.1 | 0.76 | 0.12 | 1.29 | 0.66 | 0.77 |
| Exp. No.2 | 2.40 | 0.68 | 2.91 | 1.43 | 1.79 |
| Exp. No.3 | 1.90 | 0.47 | 1.29 | 1.30 | 1.40 |

| Trf X2 |  |  |  |  | (%) |
| --- | --- | --- | --- | --- | --- |
| Add. factor: | mCherry | p53-WT | p53-R175H | MDM2 | MDM4 |
| Exp. No.1 | 0.01 | 0.00 | 0.04 | 0.01 | 0.01 |
| Exp. No.2 | 0.02 | 0.01 | 0.25 | 0.03 | 0.06 |
| Exp. No.3 | 0.12 | 0.01 | 0.16 | 0.04 | 0.09 |

*Nakagawa et al.*, Supplemental Table 2

Reprogramming efficiency of PBMCs using synthetic RNAs

|  | Additional factor (%) | | | |
| --- | --- | --- | --- | --- |
| PBMC lot. No. | d2EGFP | MDM4-WT | MDM4-S367A | MDM4-S367D |
| HHU20220825 | 0.013 | 0.073 | 0.094 | 0.062 |
| HHU20221011 | 0.012 | 0.052 | 0.080 | 0.08 |
| HHU20220519 | 0.006 | 0.059 | 0.101 | 0.071 |
| HHU20220809 | 0.035 | 0.153 | 0.114 | 0.116 |
| HHU20220830 | 0.027 | 0.055 | 0.068 | 0.075 |
| HHU20221004 | 0.102 | 0.143 | 0.220 | 0.164 |
| HHU20220317 | 0.013 | 0.089 | 0.118 | 0.106 |
| HHU20220607 | 0.002 | 0.001 | 0.009 | 0.003 |

Correspondence between PBMCs and lot numbers shown in Figure 2d:

PBMC1: HHU20221011

PBMC2: HHU20220607

PBMC3: HHU20220809

Donor information for PBMC

| PBMC lot. No. | Ethnicity | Age | Gender | ABO/Rh |
| --- | --- | --- | --- | --- |
| HHU20220825 | African/American | 48 | Male | O/Pos |
| HHU20221011 | Caucasian | 29 | Male | O/Pos |
| HHU20220519 | Caucasian | 32 | Female | O/Pos |
| HHU20220809 | African/American | 29 | Female | B/Pos |
| HHU20220830 | Caucasian | 29 | Female | O/Pos |
| HHU20221004 | Hispanic | 42 | Male | O/Pos |
| HHU20220317 | African/American | 41 | Female | O/Pos |
| HHU20220607 | Hispanic | 43 | Female | O/Pos |
